# CDK inhibitors promote neuroblastoma cell differentiation and increase sensitivity to retinoic acid—a promising combination strategy for therapeutic intervention

**DOI:** 10.1038/s41420-025-02637-z

**Published:** 2025-08-02

**Authors:** Olia Shokraie, Larissa Lechermeier, Pia Bordihn, Philipp Kaps, Steffen Möller, Anna Sophie Schulz, Björn Schneider, Dirk Koczan, Samira Khanipour Roshan, Holger N. Lode, Carl-Friedrich Classen, Olga Hahn, Sascha Troschke-Meurer, Claudia Maletzki

**Affiliations:** 1 University Children’s Hospital, Rostock University Medical Center, University of Rostock, Rostock, Germany; 2 Institute of Clinical Chemistry and Laboratory Medicine, Rostock University Medical Center, University of Rostock, Rostock, Germany; 3 Department of Internal Medicine — Clinic and Polyclinic for Hematology, Hemostaseology, Oncology, Stem Cell Therapy and Palliative Medicine, Rostock University Medical Center, University of Rostock, Rostock, Germany; 4 Institute of Pathology, Rostock University Medical Center, University of Rostock, Rostock, Germany; 5 Department of Immunology, Rostock University Medical Center, University of Rostock, Rostock, Germany; 6 Department of Pediatric Hematology and Oncology, University Medicine Greifswald, Greifswald, Germany; 7 Institute of Cell Biology, Rostock University Medical Center, University of Rostock, Rostock, Germany

**Keywords:** CNS cancer, Apoptosis

## Abstract

The rarity of recurrent somatic mutations poses a challenge for the targeted treatment of neuroblastoma (NB). Differentiation therapy is an encouraging prospect, with cyclin-dependent kinase inhibitors (CDKis) representing a promising avenue for promoting NB differentiation. This study investigated three CDKis (abemaciclib, fadraciclib, and dinaciclib) alone or combined with retinoic acid (RA) to assess the effects on morphology, growth, gene expression, and the induction of immunogenic cell death in NB cell lines with (LAN-1 and CHLA-90) and without (CHLA-172) *MYCN* amplification. All cell lines demonstrated sensitivity to CDK inhibition. Notably, low-dose abemaciclib promoted cellular differentiation, as evidenced by the emergence of stromal-like morphological features and upregulation of the differentiation markers STMN4 and ROBO2. Treatment with abemaciclib or fadraciclib led to the upregulation of calnexin and holocytochrome C, which are part of the global stress response, along with the protein p27, which arrests the cell cycle. Molecularly, CDKis sensitivity correlated with an increased *CDK4*-specific copy number, along with a partial deletion of *CDKN2a* in two cases (LAN-1, CHLA-172). The addition of RA augmented the effects of the monotherapy, particularly in LAN-1 cells, in both 2D and 3D culture, and both treatments triggered immunogenic cell death, evidenced by calreticulin translocation. Transcriptomic analysis of LAN-1 and CHLA-90 cells revealed that genes deregulated by monotherapy (fadraciclib or RA) were re-regulated in the presence of the second drug. Combination therapy significantly downregulated *CRABP2* and *CYP26B1*, both of which are involved in RA metabolism and its degradation. Furthermore, *CCNE2*, *MYBL2*, and *MCM4* were strongly suppressed in the fadraciclib/RA combination, confirming the induction of cell cycle arrest. CDKi treatments promote NB differentiation via ER stress, with cytotoxicity enhanced by RA co-treatment. This may increase NB immunogenicity and support immunotherapy eligibility.

## Introduction

Neuroblastoma (NB) is one of the most prevalent extracranial solid tumors, accounting for approximately 5.5% of all malignancies in children and adolescents. As reported by the German Childhood Cancer Registry, 90% of all newly diagnosed patients are under the age of six [1]. Despite advances in intensive multimodal therapies, patient survival remains a significant challenge, with high rates of refractoriness, progression, recurrence, and malignancy [2]. It is, therefore, evident that more effective therapeutic agents are required to achieve better outcomes in these patients. One strategy in cancer therapy is the induction of differentiation in cancer cells. This idea was first introduced in Pierce’s 1959 study of teratomas, revealing that although cancer cells mimic the process of tissue renewal, their differentiation potential differs from that of tissue stem cells [3]. Applying this concept suggests that using differentiation-inducing reagents promotes cancer cells maturation and revert the malignant cells to their cell of origin—a well-differentiated precursor—or a benign phenotype. As a result, tumor cell proliferation and aggressiveness are reduced, thereby enhancing susceptibility to therapeutic interventions. Accordingly, this approach has been applied to several cancers including NB [4, 5], particularly in high-risk patients. In NB cells, isotretinoin (retinoic acid, RA) is utilized as a reagent to induce cell differentiation and inhibit tumor growth [6] which has been a standard treatment for many years [7]. Although RA has been demonstrated to have a minor effect on long-term overall survival, it is still employed in conjunction with the GD2-specific antibody dinutuximab [8] and the post-consolidating setting [9]. Besides, molecular alterations that drive disease progression may offer new therapeutic targets. Cyclin-dependent kinases (CDKs) are considered promising targets for cancer therapy due to their pivotal role in regulating the cell cycle [10]. The CDKs 1, 2, 4, and 6 contribute to cell cycle progression [11, 12]. In addition, several so-called transcription-associated CDKs (i.e., CDK7, CDK8, CDK9, and CDK12/13) regulate the activity of RNA polymerase II, from its binding to the promoter to the recycling of the polymerase [10, 12]. Therefore, inhibiting CDKs disrupts the cell cycle of cancer cells. Two well-known CDK inhibitors (CDKis) are abemaciclib and fadraciclib, with the former primarily targeting CDK4/6 and the latter targeting CDK2/9 [11, 13–15].

In NB, *CCND1*, *CDK4*, and *CDK6* overexpression contribute to the undifferentiated phenotype, associated with a poor prognosis [16, 17]. Accordingly, CDKis possess therapeutic potential in preclinical NB models [6, 18]. CDK2 targeting triggered the killing of *MYCN*-amplified NB cells *via* impaired phosphorylation of the retinoblastoma protein (Rb) [19]. A recent study reported synergistic effects after combined CDK4/6 and PI3K blockade by inducing G1 cell cycle arrest [20]. These data confirm that CDK inhibition exerts anti-tumor effects by inducing cell cycle arrest, promoting apoptosis and necrosis, and finally reducing metastasis [6, 12]. In a clinical environment, one study already reported stable disease upon CDK4/6 blockade in pediatric cancer patients, 50% of whom suffered from NB [21]. However, a systematic analysis within the Pediatric Precision Oncology Registry (INFORM, [22]) failed to show improved overall survival of patients receiving matching targeted CDKi treatment, raising the need for interventional biomarker-driven combination approaches.

In this study, we utilized a CDK signature-driven molecular treatment strategy, applying three different CDKis alone or in combination with RA to promote differentiation-driven NB cell death. Low-dose single-agent CDK inhibition induced differentiation in both *MYCN*-amplified and non-amplified NB cells, resulting in p27-driven cell cycle arrest. The combination treatment synergistically enhanced cell stress and ultimately induced cell death.

## Results

### CDKi treatment triggers NB cell differentiation and impairs viability

**Fig. 1:**
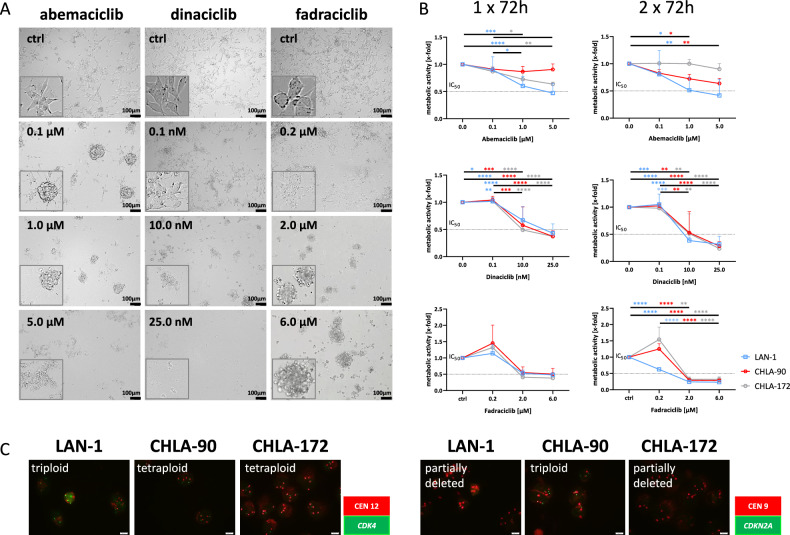
Dose response curve and molecular analysis. **A**, **B** Sensitivity of NB cells (LAN-1, CHLA-90, and CHLA-172) to abemaciclib, dinaciclib, and fadraciclib was determined after 1 × 72 h and 2 × 72 h. Cells were treated with increasing concentrations of CDKis and metabolic activity was measured using the CCK-8 reagent. **A** Representative images of LAN-1 cells after 1 × 72 h. Error bars: 100 µm. **B** Metabolic activity was quantified after the treatment by normalization to control. Mean + SD; *n* = 3 biological replicates. Two-way ANOVA (Tukey’s multiple comparisons test). *p < 0.05; ** *p* < 0.01; ***p < 0.001; ****p < 0.0001. **C** Cyto-FISH. Cytospins of NB cell lines were stained with the SPEC CDK4/CEN 12 Dual Color Probe or SPEC CDKN2A /CEN9 Dual Color Probe to check for gene-specific amplification of *CDKN2A* and *CDK4*. The red spots indicate the centromers and the green spots indicate the specific gene. Visualization was carried out with the fluorescence microscope Olympus BX53. Original magnification ×1000.

NB cells LAN-1, CHLA-90, and CHLA-172 were exposed to increasing concentrations of CDKis, and the metabolic activity was measured after 72 h and 2 × 72 h (Fig. 1). Light microscopy revealed morphology and sensitivity changes compared to untreated controls. Low-dose abemaciclib (0.1 µM) induced stromal-like features in the cells large flat cytoplasm and strong adherence, suggesting glial lineage fate. Such morphological signs of differentiation were not seen after dinaciclib and fadraciclib, which appeared to be more cytotoxic (Fig. 1A, supplementary Fig. 1). Dose curve analysis confirmed the morphological observations. As shown in Fig. 1B, longer treatment led to stronger effects; thus, subsequent experiments were conducted with the 2 × 72 h treatment period.

All cell lines showed chromosome 12 polysomy with centromere gains, indicating *CDK4* amplification (Fig. 1C). LAN-1 and CHLA-172 had partial *CDKN2A* deletion, whereas CHLA-90 showed *CDKN2A* amplification (Fig. 1C, left).

### Combination therapy of CDKis and RA synergistically boosts cell death

**Fig. 2:**
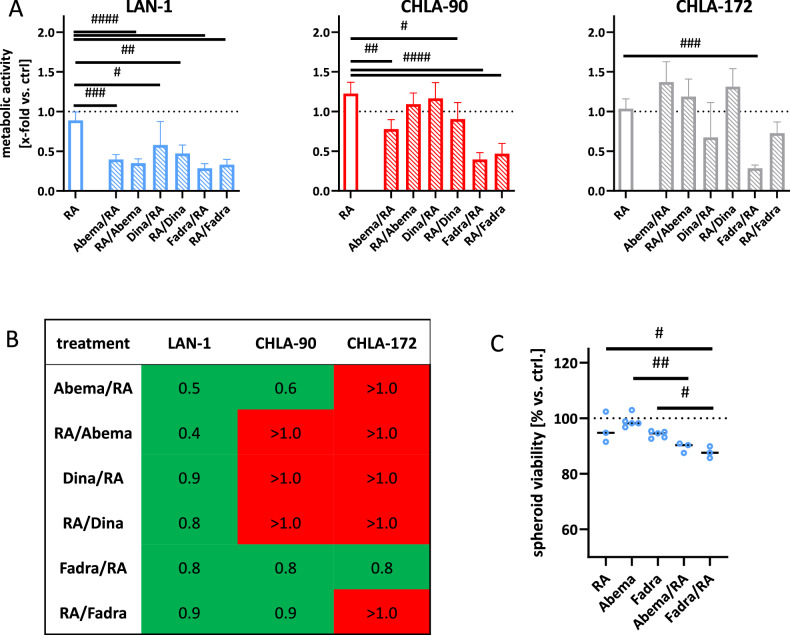
Combination approach. **A** Two treatment settings were tested on NB cells (LAN-1, CHLA-90, and CHLA-172): CDKi followed by RA and the other way around. Metabolic activity after treatment was quantified by normalization to control. Mean + SD; *n* = 3 biological replicates. Two-way ANOVA (Tukey’s multiple comparisons test). ^#^*p* < 0.05; ^##^*p* < 0.01; ^###^*p* < 0.001; ^####^*p* < 0.0001 vs. RA monotherapy. **B** Bliss independence calculation. CI < 1 synergistic; CI = 1 additive; CI > 1 antagonistic. **C** 3D-spheroid viability assessment using 3D-Glo assay. Individual values of single spheroids are shown, including the median; *n* = 3 biological replicates. One-way ANOVA (Tukey’s multiple comparisons test). ^#^*p* < 0.05; ^##^*p* < 0.01; vs. monotherapy. **A**–**C** Doses: RA (all cell lines): 1.5 µM; LAN-1 abemaciclib: 0.2 µM; dinaciclib (all cell lines): 10 nM; fadraciclib: 0.4 µM; CHLA-90/CHLA-172: abemaciclib: 1.0 µM: fadraciclib: 1 µM.

CDKis were then combined with the differentiation agent RA to investigate potential synergistic effects. RA was applied at doses within the range of pharmacokinetics in children (1.5 µM). Two different approaches were used: (I) CDKi first, RA second, and (II) RA first, CDKi second. (I) was more effective than (II) and synergistically enhanced the antitumor effects of the monotherapy in all cell lines (Fig. 2A, B). Notably, the sequential timing of fadraciclib application had little effect on the response in all three cell lines, as both regimens resulted in effective growth inhibition (Fig. 2A, right, and B). In CHLA-90 cells, sequential treatment with abemaciclib and RA demonstrated a synergistic effect compared to monotherapies, highlighting the superior efficacy of the selective CDK inhibitor abemaciclib over the broader-acting CDK inhibitor dinaciclib. Consequently, we focused on sequential CDKi and RA therapies in subsequent experiments.

First, we validated the antitumor effects of the CDKi-based combination strategy in 3D spheroid models using LAN-1 cells (Fig. 2C). After two treatment cycles, spheroid viability was significantly reduced by the combination therapies, with statistically significant effects observed for both abemaciclib and fadraciclib combinations (*p* < 0.01 and *p* < 0.05, respectively).

### CDKi-induced differentiation of NB cells is preserved in the combination

**Fig. 3:**
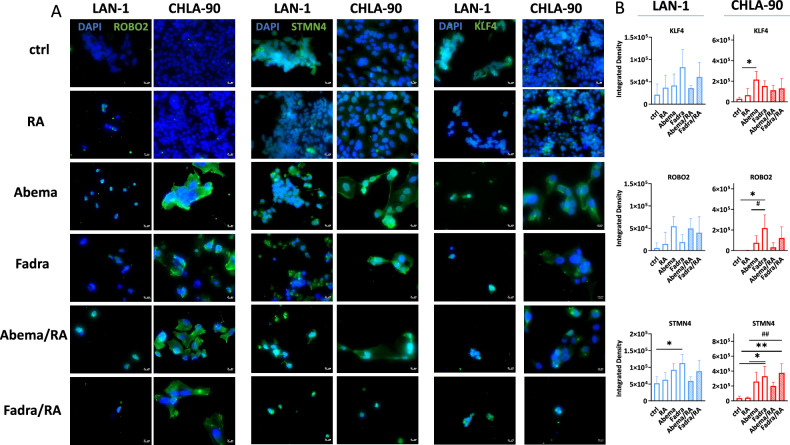
Immunofluorescence for detection and quantification of differentiation and stemness markers ROBO2, STMN4, and KLF4. NB cells (LAN-1, CHLA-90, and CHLA-172) were stained with respective antibodies to detect target proteins (green), nuclei were stained with DAPI (blue). Images were taken on a Zeiss AxiovertA.1 Microscope. **A** Representative microscopic images of NB cells, taken on a Zeiss AxiovertA.1 Microscope. **B** Quantification of ROBO2, STMN4, and KLF4. Mean + SD; *n* = 3 biological replicates. One-way ANOVA (Tukey’s multiple comparisons test). *p < 0.05; **p < 0.01; ^#^*p* < 0.05; ^##^*p* < 0.01. **A**, **B** Doses: RA (all cell lines): 1.5 µM; LAN-1 abemaciclib: 0.2 µM; fadraciclib: 0.4 µM; CHLA-90/CHLA-172: abemaciclib: 1.0 µM: fadraciclib: 1 µM.

To determine whether observed differentiation correlates with protein level changes, differentiation markers ROBO2 and STMN4 and the stemness marker KLF4 were studied. Our analysis revealed an upregulation of ROBO2, STMN4, and KLF4 proteins in LAN-1 and CHLA-90 cells in all settings (Fig. 3). In LAN-1, significant upregulation occurred only for STMN4 after fadraciclib monotherapy. In CHLA-90, KLF4 was significantly higher with abemaciclib and ROBO2 was significantly higher after treatment with fadraciclib. Moreover, STMN4 was significantly upregulated in CHLA-90 cells by fadraciclib mono- and combination therapy compared to the control and RA groups.

### CDKi mono- and combination treatment triggers stress in differentiated NB cells

**Fig. 4:**
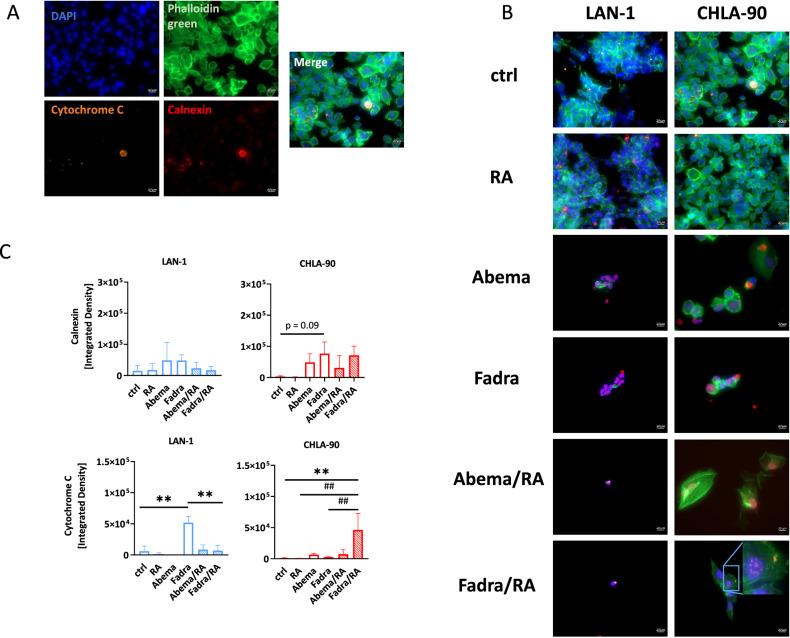
Immunofluorescence for detection and quantification of cytochrome C and calnexin. **A**, **B** NB cells (LAN-1, CHLA-90) were stained with respective antibodies to determine the subcellular localization of cytochrome C (green), and calnexin (red). The cytoskeleton was visualized using phalloidin green, nuclei were stained with DAPI (blue). Images were taken on a Zeiss AxiovertA.1 Microscope. **A**, **B** Representative single channel and merged images. **C** Quantification of cytochrome C and calnexin intensity. Mean + SD; *n* = 3 biological replicates. One-way ANOVA (Tukey’s multiple comparisons test). **p < 0.01 vs. ctrl; ^##^*p* < 0.01 vs. monotherapy. (B, C) Doses: RA (all cell lines): 1.5 µM; LAN-1 abemaciclib: 0.2 µM; fadraciclib: 0.4 µM; CHLA-90: abemaciclib: 1.0 µM: fadraciclib: 1 µM.

Since CDKi induced endoplasmic reticulum (ER) stress in glioblastoma cells, characterized by increased calnexin abundance and cytochrome C redistribution [23], we investigated similar mechanisms in NB cells. Calnexin, an ER chaperone, increased after CDKi monotherapy compared to controls (Fig. 4). The addition of RA did not increase ER stress and calnexin levels remained comparable to CDKi monotherapy. Evaluation of cytochrome C yielded cell line-specific results (Fig. 4). In LAN-1 cells, fadraciclib upregulated cytochrome C, but remained at baseline levels in the combination. In CHLA-90 cells, the fadraciclib/RA combination led to a cytochrome C accumulation in the mitochondria (Fig. 4B inbox magnification). Overall, the ER stress induced by CDKi monotherapy was superseded by the cytotoxicity after the addition of RA, and the low number of residual cells after combined CDKi/RA treatment compromised data interpretation.

### CDKi mono- and combination treatment induces G1 cell cycle phase arrest and triggers necrotic and immunogenic cell death

**Fig. 5:**
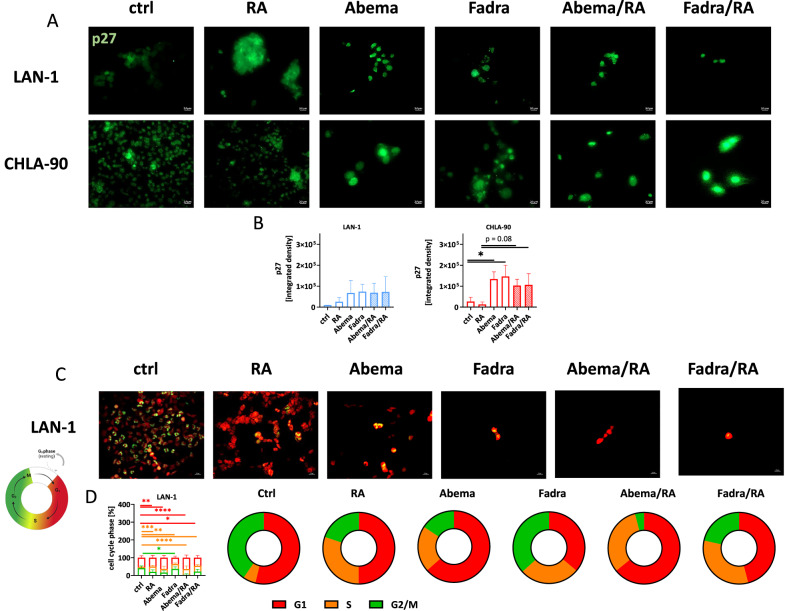
Detection of cell cycle arrest by p27 immunofluorescence and cell death by flow cytometry. **A**, **B** p27 was detected upon staining with an Alexa Fluor® 488 anti-p27/Kip1 Antibody. **A** Representative microscopic images of NB cells (LAN-1, CHLA-90), taken on a Zeiss AxiovertA.1 Microscope. **B** p27 quantification. Mean + SD; *n* = 3 biological replicates. One-way ANOVA (Tukey’s multiple comparisons test). *p < 0.05. **C**, **D** Cell cycle analysis of lentivirally-transduced LAN-1 cells. **C** Visualization of cell cycle phases by fluorescence microscopy. **D** Quantification of cell cycle phases. *n* = 3 biological replicates. Two-way ANOVA (Tukey’s multiple comparisons test). *p < 0.05; **p < 0.01; ***p < 0.001; ****p < 0.0001 vs. ctrl. **A**–**D** Doses: RA (both cell lines): 1.5 µM; LAN-1 abemaciclib: 0.2 µM; fadraciclib: 0.4 µM; CHLA-90: abemaciclib: 1.0 µM: fadraciclib: 1 µM.

**Fig. 6:**
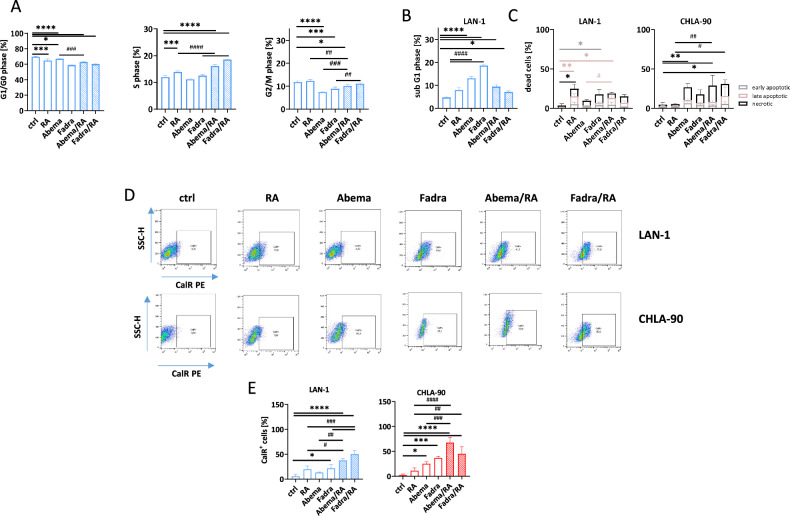
Detection of cell cycle changes and treatment-related CalR translocation. **A**, **B** Two-step cell cycle analysis: Cells were counted, adjusted to equal numbers, lysed, and stained with DAPI prior to measurement. DNA content was measured using the NucleoCounter® NC-3000™ system. Mean + SD; *n* = 3 biological replicates. One-way ANOVA (Tukey’s multiple comparisons test). *p < 0.05; ***p < 0.001; ****p < 0.0001 vs. ctrl. ^##^*p* < 0.01; ^###^*p* < 0.001; ^####^*p* < 0.0001 vs. monotherapy. **C** Apoptosis/necrosis assay. Flow cytometry was done to quantify the number of dead cells after 2 × 72 h treatment. Early apoptotic cells: Yo-Pro-1^+^/PI^-^, late apoptotic cells: Yo-Pro-1^+^/PI^+^, necrotic cells: Yo-Pro^-^1^-/^PI^-^. *n* = 3 biological replicates. One^-^way ANOVA (Tukey’s multiple comparisons test). *p < 0.05; **p < 0.01; ***p < 0.001 vs. ctrl; ^#^*p* < 0.05; ^##^*p* < 0.01; ^###^*p* < 0.001 vs. monotherapy. **D**, **E** Immunogenic cell death analysis of NB cells ^(^LAN-1, CHLA-90) was done by flow cytometry using PE anti-calreticulin antibody. **D** Representative flow cytometry plots of LAN-1 and CHLA-90 cells. **E** Quantitative analysis. **A**–**E** Doses: RA (both cell lines): 1.5 µM; LAN-1 abemaciclib: 0.2 µM; fadraciclib: 0.4 µM; CHLA-90: abemaciclib: 1.0 µM: fadraciclib: 1 µM; Mean + SD; *n* = 3 biological replicates. One-way ANOVA (Tukey’s multiple comparisons test). *p < 0.05; ***p < 0.001; ****p < 0.0001 vs. ctrl; ^#^*p* < 0.05; ^##^*p* < 0.01; ^###^*p* < 0.001; ^####^*p* < 0.0001 vs. monotherapy.

Our findings confirmed NB cell differentiation upon treatment, but also revealed enhanced cell stress and cytotoxicity in the combination, warranting detailed investigation of underlying mechanisms (Fig. 5). All regimens led to an upregulation of p27 compared to controls, indicating G1 cell cycle arrest (Fig. 5A, B). This effect was primarily driven by CDKis, reaching significance in CHLA-90 cells following abemaciclib and fadraciclib treatment, and remained high in the combination. These results were further validated by monitoring G1 and S/G2/M phases in lentivirally transduced LAN-1 cells (Fig. 5C, D). Abemaciclib-induced G1 cell cycle arrest persisted in the combination treatment (*p* < 0.0001 vs. ctrl). Fadraciclib, either alone or in combination caused a modest shift in cell cycle phases, increasing the proportion of cells in the S-phase after monotherapy – an effect amplified in the combination. These changes were likewise detectable via flow-based cell cycle analysis on LAN-1 cells (Fig. 6A). Both combination regimens led to a significant induction of cells in S phase. Also, the proportion of cells in G1/G2 phases differed between controls and mono- or combination treatments, with a trend towards most changes after combination with abemaciclib or fadraciclib. However, in most cases, only a few residual cells were detectable, indicating massive induction of cell death. Indeed, the proportion of cells with sub G1-phase significantly increased (Fig. 6B). Highest proportions of cells with sub G1 phase were detected after CDKi and RA/CDKi combination treatment (Fig. 6B). Accompanying, apoptosis/necrosis confirmed cell death induction (Fig. 6C). RA monotherapy significantly increased the amount of late apoptotic and necrotic cells in LAN-1 cells, but had no significant impact on cell death in CHLA-90 cells. CDKis also induced necrosis in both cell lines, with a significant increase in CHLA-90 cells after abemaciclib (*p* < 0.01 vs. ctrl). The combination of RA with abemaciclib and fadraciclib significantly enhanced necrotic effects in CHLA-90 cells, but not in LAN-1 cells (Fig. 6C). The number of necrotic cells significantly increased when RA was combined with abemaciclib or fadraciclib. Assessing immunogenicity via CalR translocation using flow cytometry (Fig. 6D, E) showed that all treatments resulted in CalR translocation in both cell lines compared to controls. All mono- and combination treatments resulted in CalR translocation, notably in both cell lines, compared with untreated controls (Fig. 6E).

In summary, the RA/CDKi combination boosts the antitumor effects of the monotherapy primarily via induction of necrotic and immunogenic cell death.

### Transcriptional changes in cell cycle and differentiation-associated genes after CDKi mono- and combination treatment

**Fig. 7:**
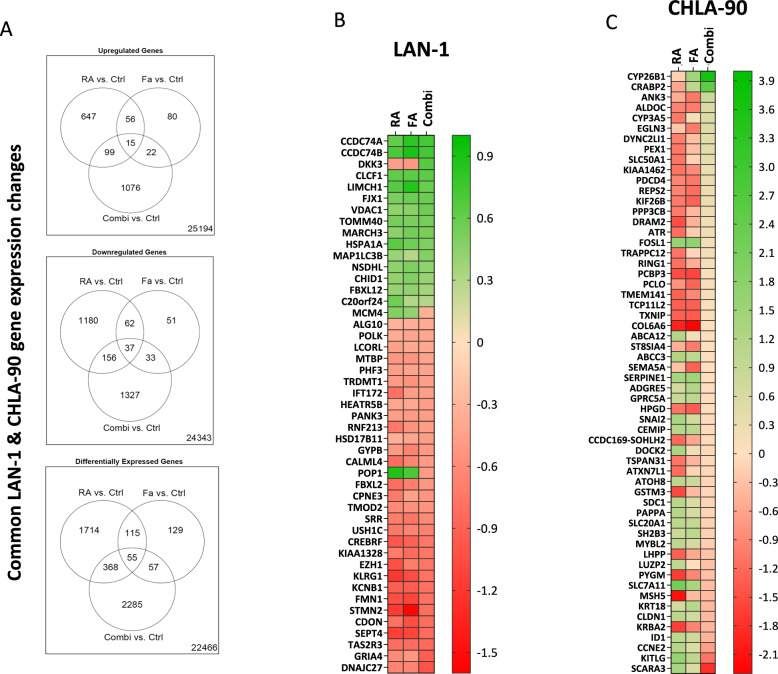
Microarray analysis to confirm treatment-induced changes in LAN-1 and CHLA-90 cells on a transcriptional level. **A** Venn diagrams illustrate the number of genes that were differentially expressed after receiving RA (RA vs. Ctrl), fadraciclib (Fa vs. Ctrl), and the combination (Combi vs. Ctrl). Therefore, the data from the two cell lines LAN-1 and CHLA-90 were combined to eliminate the effects of the individual cell lines on the therapy. This was done to determine if the treatment itself can regulate the specific gene in both cell lines. Each circle represents the respective gene sets, with the default settings of a Benjamini–Hochberg adjusted *P* value < 0.05. **B**, **C** The 55 probe sets (mapping to 47 and 58 genes) in the center of the DEGs are depicted in the heatmap. **B** LAN-1 cells and (**C**) CHLA-90 cells.

**Fig. 8:**
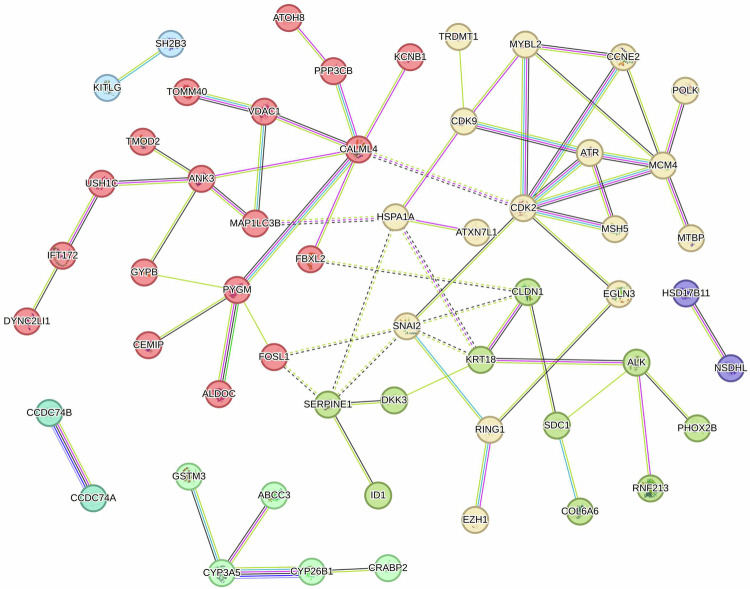
Interaction network of genes affected by the therapy in neuroblastoma. The nodes represent proteins encoded by corresponding genes, and the edges between them indicate known or predicted interactions. MCL clustering was implemented considering 1.3 inflation parameter. Every color corresponds to a cluster, inter-cluster edges are represented by dashed lines.

Finally, treatment-related transcriptional changes were determined by gene expression and functional enrichment analyses. LAN-1 and CHLA-90 cells were used for analyses, with a focus on RA and fadraciclib in mono- and combination treatment (Figs. 7, 8).

Gene expression of both cell lines was significantly affected by both mono- and combination therapies, despite their distinct genetic backgrounds. Venn diagrams (Fig. 7A) showed that numerous genes were up- or down-regulated in each regimen, with some overlap. The differentially expressed gene (DEGs) analysis identified 55 probe sets, represented as 47 genes in the heatmap (Fig. 7B). *POP1* was upregulated in monotherapies but downregulated in the combination, whereas *DKK3* showed the opposite expression pattern. However, gene enrichment analysis and STRINGdb did not reveal any specific relationship or activated signaling pathway.

Examining the gene set (Fig. 7B, Supplementary Table 1), we postulated that the combination could reverse the effects of the individual treatments. Figure 7C and Supplementary Table 2 show genes that were significantly altered by a single treatment and subsequently re-regulated under the influence of the second drug. Note that the genes in question are not necessarily present within the intersections of the gene sets depicted in the Venn diagrams (Fig. 7A), because a comparison of the combination vs. the control was not required to display significant differences. Instead, we focused on comparisons between fadraciclib mono- and combination treatment, filtering for log fold changes in different directions. Similarly, two upregulated genes, *CRABP2* and *CYP26B1* involved in RA metabolism and degradation, were altered by the combination. These genes were slightly downregulated by RA alone, but showed a fourfold change in the combination.

Subsequently, we combined the two gene sets (47 and 56) and analyzed the interaction network using STRINGdb (Fig. 8). To refine our analysis, we included additional genes: inhibitor targets such as *CDK2* and *CDK9*, neuroblastoma-associated genes, *ALK* and *PHOX2B*, along with the two upregulated genes from the combination, *CRABP2* and *CYP26B1*. Using MCL clustering [24], we found four major clusters (yellow, red, mustard green, and green) of gene interactions. Several genes in the yellow and green clusters indicated important regulatory changes. In the yellow cluster, cell cycle regulators such as *CCNE2*, *MYBL2*, and *MCM4* were strongly suppressed in the combination, confirming that this approach induced cell cycle arrest and exerted a stronger inhibition of tumor cell proliferation compared to either treatment alone (Supplementary Table 1 and 2, Supplementary Fig. 2). Conversely, *CRABP2* and *CYP26B1* clustered in green displayed a significant upregulation in the combination, with log fold changes of 2.48 and 3.66, respectively. Finally, *PYGM* (muscle glycogen phosphorylase; providing energy for proliferating cells) in the red cluster was found to be downregulated in the combination.

Several genes, including *MSH5*, *ATR*, and *FBXL2*, displayed a slight recovery to baseline levels in combination; however, they remained downregulated. This indicates a suppression of DNA repair and proteasomal degradation pathways, adversely affecting cancer proliferation and survival, leading to the accumulation of damaged proteins and increased stress within cancer cells.

## Discussion

In this study, we investigated CDKis as potential combination partners for RA-based treatment to induce differentiation of NB cells. The CDKis included FDA-approved abemaciclib and near-clinical candidates dinaciclib and fadraciclib. RA-based differentiation therapies are effective only in a subset of NB patients but are clinically implemented for treating HR^+^/HER2^−^ advanced breast cancer [25, 26]. In preclinical tumor models, CDKis have demonstrated efficacy in inducing terminal differentiation and apoptosis of both primary and metastatic NB tumors [6, 27], encouraging further exploration of their potential for enhancing NB treatment options through synergistic effects.

Our previous studies in head and neck cancer identified copy number alterations in *CDKN2A* and/or *CDK4* as predictive biomarkers [28] and molecular correlates of response to abemaciclib. In this study, NB cell lines with distinct morphological phenotypes (i.e. LAN-1: adrenergic; CHLA-90: mesenchymal-like; CHLA-172: mesenchymal) showed *CDK4* amplification and partial *CDKN2A* loss. Despite these shared molecular features, cell line-specific differences were observed. LAN-1 cells were the most sensitive to abemaciclib with IC_50_ values below plasma levels. In contrast, CHLA-90 and CHLA-172 cells showed a dose-dependent reduction in metabolic activity, indicating a slightly delayed response. This finding supports the hypothesis that these mesenchymal-like cells may rely more on stress-response pathways than on rapid proliferation for survival, contributing to their distinct treatment response. Furthermore, we propose that *CDK4* amplification and/or *CDKN2A* loss serve as potential biomarkers for CDKi-based combination strategies, independent of the cell growth kinetics. The well-established oncogenic role of cell cycle proteins within the cyclin D/CDK4/CDK6/RB network in NB [29] contrasts with the variability in the *CDKN2A* status (i.e. either deletions or amplifications), and underscores the need for molecular-driven personalization.

Although molecular biomarkers for dinaciclib and fadraciclib have not yet been proposed, the complex alterations in cell cycle-associated genes in primary and metastatic NB cases and the relevance of alterations in individual oncogenes suggest a potential application for these CDKis. In initial screenings, low-dose abemaciclib induced morphological differentiation in NB cells, while dinaciclib and fadraciclib were predominantly cytotoxic, with fadraciclib displaying biphasic responses. At ultra-low doses, cells exhibited increased metabolic activity, a common compensatory stress response, where cells adapt to low-dose treatment by shifting their metabolism (e.g., upregulating glycolysis or oxidative phosphorylation) to enhance survival. This mild stress can activate key survival pathways, including NF-κB and PI3K/AKT/mTOR, promoting cell survival and proliferation [30, 31]. However, at higher doses, this stress-response becomes cytotoxic, ultimately leading to cell death. Despite these individual differences, it is worth mentioning that all three inhibitors effectively reduced NB cell viability, with improved efficacy following prolonged treatment, whereas RA monotherapy had no effect. Notably, combining CDK inhibitors with RA synergistically enhanced treatment outcomes and significantly impaired cell viability, especially in LAN-1 cells - cultured in both 2D and 3D spheroid models.

Mechanistically, prolonged exposure may be essential to yield sufficient inhibition of CDK activity, inducing cell cycle arrest, differentiation, or apoptosis. Consistent with our findings, Xie et al. reported abemaciclib-mediated differentiation of leukemia stem cells in vitro and in vivo, characterized by impaired self-renewal and suppressed cell proliferation [32]. Abemaciclib’s antitumor effects have been validated in multiple cancer types [33–37]. Ferguson and colleagues showed that palbociclib inhibited NB proliferation and promoted differentiation to a more mature neuronal phenotype, similar to abemaciclib [38]. Our study also revealed that gene expression and protein abundance of differentiation (*STMN4* and *ROBO2*) and stemness-associated (*KLF4*) molecules significantly changed after abemaciclib and fadraciclib mono- and combination treatment with RA. Although these change may be a interpreted as a therapy-induced stress and the accumulation of stem cell-like properties [39], these changes cumulatively drive differentiation critical for tumor control. Thus, combining CDK4/6 inhibitors such as abemaciclib or palbociclib with RA represents an innovative strategy to improve current NB treatment.

Consistent with these findings, our microarray analysis revealed a significant effect on key genes involved in differentiation, proliferation, and apoptosis. Specifically, *ID1* (inhibitor of differentiation/DNA binding) and *POP1* were downregulated, while *DKK3*, *ALDOC*, *CRABP2*, and *KRT18* were upregulated, collectively promoting differentiation and suppressing proliferation. While the upregulation of *DKK3* and *ALDOC* enhanced differentiation, the downregulation of *ID1* may reduce the oncogenic potential, reinforcing the tumor-suppressive effects of the treatment, as demonstrated before [40].

*POP1* plays a role in tRNA processing. Its upregulation in monotherapy may indicate a compensatory response for RNA processing under stress, but combination treatments inhibit RNA processing, affecting cell growth and proliferation [41, 42]. *DKK3* inhibits the Wnt/β-catenin pathway and acts as a tumor suppressor or oncogene [43], when upregulated in breast, ovarian, colon, and pancreas cancers, then it is associated with poor outcomes. Furthermore, DKK3 may induce cell cycle arrest and apoptosis via the Wnt/β‑catenin pathway, and reduce cell proliferation and invasiveness in lung adenocarcinoma [44]. According to our study, *DKK3* upregulation in the combination may indicate Wnt/β‑catenin pathway modulation, thereby inhibiting cell proliferation and inducing differentiation or apoptosis.

The joint upregulation of *MCM4* and *MAP1LC3B* in the combination indicates increased cell cycle regulation and autophagy. Hence, effective regulation of proliferation and differentiation through the RA/CDKi combination may force cells into the non-proliferative state, improving long-term outcomes.

A recent study indicated that fadraciclib targeted CDK9 in NB cells, suppressed *MYCN* transcription, and induced growth arrest, resulting in increased sensitivity to apoptosis when combined with CDK2 inhibition [45].

Flow cytometry showed varying levels of late apoptotic and/or necrotic cells following mono- and combination therapy with RA, abemaciclib, and fadraciclib, observing the highest efficacy for the RA/abemaciclib combination. Abemaciclib increases early apoptotic cells in GBM, while dinaciclib predominantly induces necrosis [46]. Another study in lung and breast cells demonstrated an atypical cell death called methuosis accompanied by cytoplasmic vacuole formation after abemaciclib, suggesting that cell death induction was not mediated through ER stress, apoptosis, or necrosis [47].

Assessment of the stress markers calnexin and cytochrome C revealed a dynamic regulation pattern, reflecting cellular responses depending on the cell line, the inhibitor type, and the experimental setting. Further analysis showed p27 overexpression in RA-CDKi-based combination, confirming G1 cell cycle arrest [48]. The same outcome was observed in myeloma cells after inhibiting CDK4/6 activity. This inhibition suppressed gene expression during the early G1 phase, thereby preventing *Skp2* expression, essential for p27 degradation, as well as blocking *CDK2* and *cyclin A* expression, crucial for the S phase transition [49].

In summary, targeting CDKs alone or in combination with RA promotes cellular differentiation towards a mature neuronal phenotype, induces p27-mediated cell cycle arrest, and triggers immunogenic cell death. These findings provide a basis for refining CDKi/RA-based combination therapies and exploring their potential in immunotherapy.

## Materials and methods

### Cell culture

Long-term established human NB cell line LAN-1 was commercially obtained from the American Type Culture Collection (ATCC, Manassas, VA, USA; ACC 655, 09/2009) and the other cell lines CHLA-90 and CHLA-172 were provided by the childhood cancer repository (Texas Tech University Health Sciences Center). LAN-1 was cultured in RPMI ( + 10% FCS, L-glutamine (2 mmol/l), antibiotics (100 U/ml penicillin/100 μg/ml streptomycin, Pan Biotech, Aidenbach, Germany). For CHLA-90 and CHLA-172, IMDM was used ( + 20% FCS, L-glutamine (4 mmol/l), antibiotics (30 U/ml penicillin/30 μg/ml streptomycin), Insulin-Serin transferrin). All cells were incubated at 37 °C in 5% CO_2_. In some experiments, lentivirally transduced LAN-1 cells were used. Therefore, cells were transduced using the Incucyte® Cell Cycle Lentivirus Reagents (Sartorius, Göttingen, Germany). Cell Cycle Lentivirus Reagents take advantage of cell cycle dependent changes in the expression patterns of Geminin and CDT1. By linking fluorescent proteins TagGFP2 and mKate2 (green/red), or TagGFP2 and TagRFP (green/orange) to fragments of Geminin and CDT1, the G1 and S/G2/M phases can be visualized. Cell cycle analysis was done by fluorescence microscopy.

### Drugs and targeted substance

Isotretinoin (RA, 1.5 µM) and the CDKis (all from Selleckchem, Munich, Germany) abemaciclib (0.2 or 1 μM), dinaciclib (10 nM), and fadraciclib (0.4 and 1 µM) were used. The substances were applied at doses below the IC_50_ determined in the preliminary dose-finding study.

### Metabolic activity assay

Cell viability was assessed using a CCK-8 assay (Sigma Aldrich, St. Louis, Missouri, United States). In a 96-well plate, 10,000 cells were seeded in triplicates, incubated for 24 h, and then treated for 1 × 72 and 2 × 72 h. Thereafter, 10 μl of CCK-8 solution was added per well, and absorbance was measured at 450 nm using a microplate reader (Infinite® M200, Tecan Group, Switzerland).

### Cyto-FISH

Gene-specific amplifications were examined from 30,000 cells per cell line, fixed on a coated cytoslide (THARMAC cellspin, Wiesbaden, Germany) using SHANDON cytospin3 centrifuge cell preparation system (10 min, 700 rpm). The gene-specific staining of *CDKN2A* and *CDK4* was conducted using the ZytoLight FISH cytology implementation kit (ZytoVision, Bremerhaven, Germany) according to the manufacturer’s instructions with ZytoLight SPEC CDKN2A/CEN9 Dual Color Probe and ZytoLight SPEC CDK4/CEN12 Dual Color Probe (ZytoVision, Bremerhaven, Germany).

### Apoptosis/Necrosis Assay and Immunogenic Cell Death

To differentiate between early and late apoptotic, and necrotic cells, a Yo-Pro-1/propidium iodide (PI) assay was performed as described [50]. Early and late apoptotic cells were detected by Yo-Pro-1 or Yo-Pro-1/ PI positivity. Necrotic cells were defined as Yo-Pro-1 negative/PI positive.

Immunogenic cell death analysis was performed by staining surface-translocated calreticulin (CalR). Cells were incubated for 30 min at 4 °C with an PE-labeled anti-CalR antibody (1:50; EnzolifeSciences, Lörrach, Germany) and analyzed using a flow Cytometer (BD FACSVerse™, BD Pharmingen, San Jose, CA, USA).

### Determination of Cell Number, Cell Viability, Cell Size, & Cell Cycle Analysis

Cell cycle analysis was performed in triplicate using the NucleoCounter® NC-3000^TM^ (Chemometec, Allerod, Denmark) ‘2-Step Cell Cycle Assay’ according to the manufacturer’s instructions. DNA content was measured by DAPI staining and analyzed using FlowJo software (version 10.10.0).

### Immunofluorescence

NB cells (5000 cells/well) were seeded in IBIDI slides and subjected to two consecutive 72 h mono- and combined therapies with RA and CDKis. After fixation with paraformaldehyde (15 min, Polysciences, Germany) and washing steps, permeabilization and blocking were done with 0.5% Triton X-100 (Sigma Aldrich) and 5% BSA for 60 min. Cells were stained overnight with Alexa Fluor® 488 anti-p27/Kip1 Antibody (1:50, Novus Biologicals, Nordenstadt, Germany), Alexa 594 anti-calnexin antibody (AF18, 1:50, Santa Cruz, Dallas, Tx, USA), Alexa Fluor® 594 anti-cytochrome C (1:50, Biolegend, Heidelberg, Germany). Abundance of ROBO2 (1:50), STMN4 (1:50,), and KLF4 (1:50, all ThermoFisher Scientific, Waltham, MA USA) were evaluated by indirect staining using anti-mouse IgG (1:250, Alexa Fluor® 488 Conjugate, Cell Signaling Technology, Danvers, MA, USA) and anti-rabbit IgG (1:250, DyLight™ 488, Biolegend). In some cases, cells were stained with phalloidin green (1:50, ThermoFisher Scientific) and incubated for 20 min at room temperature. After washing, cells were stained with DAPI for 2 min and analyzed on a Zeiss AxiovertA.1 Microscope (Carl Zeiss, Oberkochen, Germany).

### Microarray analysis

Total RNA of LAN-1 and CHLA-90 cells was extracted and quantified, followed by gene expression profiling using Applied Biosystem^TM^ Clariom^TM^ S arrays (formerly Affymetrix, ThermoFisher Scientific), as described [46, 51]. Gene expression data were analyzed with the Bioconductor packages oligo [52] and limma [53]. To isolate the drug-specific effects, inter-cell line variability was adjusted using the batch effect removal function in limma. The script that performed the analysis is provided as a supplement.

### Image processing

The quantification of images was done by using the Fiji-ImageJ software as follows: Images were split into respective channels via ZEN software (Zeiss, Oberkochen). Staining intensity was determined by integrated density profiles of the same size.

### Statistics

Statistical analysis were conducted using GraphPad PRISM software, version 8.0.2 (GraphPad Software, San Diego, CA, USA). All values are reported as mean ± SD and a *p*-value of <0.05 was considered significant.

## Supplementary information

supplementary Figure 1

supplementary Figure 2

Supplementary material legends

Supplementary Table 1

Supplementary Table 2

## Data Availability

All datasets are either presented in the main manuscript or in additional supporting files.
