# Supplementary figures and images for: CDK inhibitors promote neuroblastoma cell differentiation and increase sensitivity to retinoic acid—a promising combination strategy for therapeutic intervention
Source: Cell Death Discov. 2025 Aug 2;11:363. doi: 10.1038/s41420-025-02637-z (PMC12318081; doi:10.1038/s41420-025-02637-z)

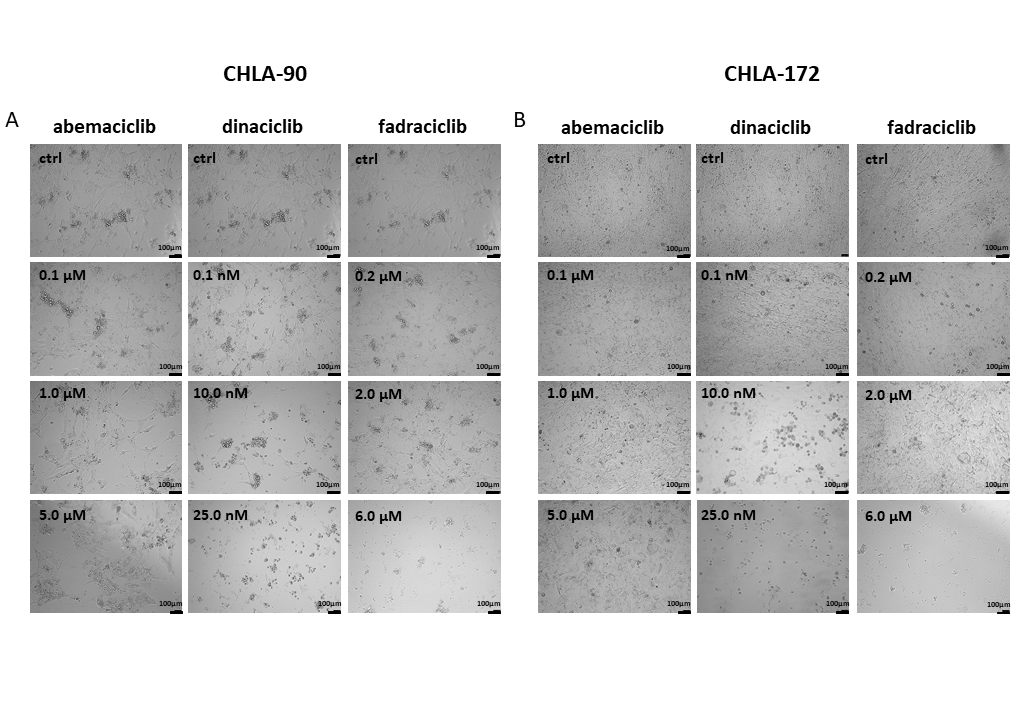

Supplement: Supplementary file 1 — supplementary Figure 1 [file 41420_2025_2637_MOESM1_ESM.tif]

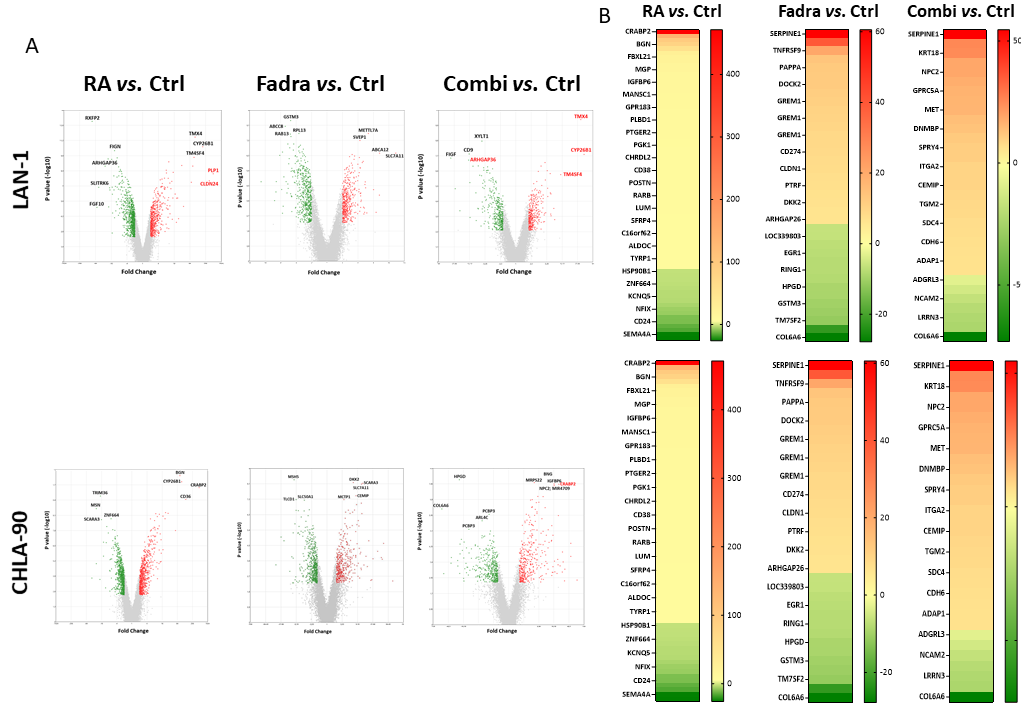

Supplement: Supplementary file 2 — supplementary Figure 2 [file 41420_2025_2637_MOESM2_ESM.tif]
