# Supplementary material for: CDK inhibitors promote neuroblastoma cell differentiation and increase sensitivity to retinoic acid—a promising combination strategy for therapeutic intervention
Source: Cell Death Discov. 2025 Aug 2;11:363. doi: 10.1038/s41420-025-02637-z (PMC12318081; doi:10.1038/s41420-025-02637-z)
Supplement: Supplementary file 3 — Supplementary material legends [file 41420_2025_2637_MOESM3_ESM.docx]

**Supplementary Figure 1: Dose response curve.** (A, B) Representative images of sensitivity of NB cells CHLA-90, and CHLA-172 to abemaciclib, dinaciclib and, fadraciclib taken after 1 x 72 h. Error bars: 100 µm.

**Supplementary Figure 2: Microarray analysis to confirm treatment-induced changes in LAN-1 and CHLA-90 cells on a transcriptional level.** (A) Volcano plot illustrating individual deregulated genes after receiving RA (RA vs. Ctrl), fadraraciclib (Fa vs. Ctrl), and the combination (Combi vs. Ctrl). (B) Heatmap representing individual genes that were either up- or downregulated by individual treatments.

**Supplementary Table 1: Genes differentially expressed in both singular treatments and in the combined treatment.**The first three columns indicate each condition including the direction of the change of expression, followed by the description of the log fold change and the adjusted P value.

**Supplementary Table 2: Genes changing their expression in the other direction upon receiving second drug.** The 77 probe sets (mapping to 56 genes) have been identified that are significantly (absolute log fold change (log FC) >1, adjusted P value < 0.05) altered upon receiving a single drug and also significantly (same criteria) counter-regulated upon the second drug.
